# Outcome measures in clinical trials of treatments for acute severe haemorrhage

**DOI:** 10.1186/s13063-018-2900-4

**Published:** 2018-10-01

**Authors:** Amy Brenner, Monica Arribas, Jack Cuzick, Vipul Jairath, Simon Stanworth, Katharine Ker, Haleema Shakur-Still, Ian Roberts

**Affiliations:** 1 Clinical Trials Unit, Department of Population Health, London School of Hygiene and Tropical Medicine, Keppel Street, London, WC1E 7HT UK; 2 Centre for Cancer Prevention, Wolfson Institute of Preventive Medicine, Queen Mary University of London, London, EC1M 6BQ UK; 3 Department of Medicine, Division of Gastroenterology, University Hospital, Western University, London, ON Canada; 4 Transfusion Medicine, NHS Blood and Transplant, Oxford, UK; 5 Department of Haematology, Oxford University Hospitals NHS Foundation Trust, Oxford, UK; 6 Radcliffe Department of Medicine, University of Oxford, and Oxford BRC Haematology Theme, Oxford, UK

**Keywords:** Blood transfusion, Clinical trial, Haemorrhage, Haemostasis, Mortality, Outcome measure, Trial methodology

## Abstract

**Background:**

Acute severe haemorrhage is a common complication of injury, childbirth, surgery, gastrointestinal pathologies and other medical conditions. Bleeding is a major cause of death, but patients also die from non-bleeding causes, the frequency of which varies by the site of haemorrhage and between populations. Because patients can bleed to death within hours, established interventions inevitably take priority over randomisation into a trial. These circumstances raise challenges in selecting appropriate outcome measures for clinical trials of haemostatic interventions.

**Main body:**

We use data from three large randomised controlled trials in acute severe haemorrhage (CRASH-2, WOMAN and HALT-IT) to explore the strengths and limitations of outcome measures commonly used in trials of haemostatic treatments, including all-cause and cause-specific mortality, blood transfusion and surgical interventions. Many deaths following acute severe haemorrhage are due to patient comorbidities or complications rather than bleeding. If non-bleeding deaths are unaffected by a haemostatic intervention, even large trials will have low power to detect an effect on all-cause mortality. Due to the dilution from deaths unaffected or reduced by the trial treatment, all-cause mortality can also obscure important harmful effects. Additionally, because the relative contributions of different causes of death vary within and between patient populations, all-cause mortality is not generalisable. Different causes of death occur at different time intervals from bleeding onset, with bleeding deaths generally occurring early. Time-specific mortality can therefore be used as a proxy for cause in un-blinded trials where bias is a concern or in situations where cause of death cannot be assessed. Urgent treatment is critical, and so post-randomisation blood transfusion and surgery are often planned before or at the time of randomisation and therefore cannot be influenced by the trial treatment.

**Conclusions:**

All-cause mortality has low power, lacks generalisability and can obscure harmful effects. Cause-specific mortality, such as death due to bleeding or thrombosis, avoids these drawbacks. In certain scenarios, time-specific mortality can be used as a proxy for cause-specific mortality. Blood transfusion and surgical procedures have limited utility as outcome measures in trials of haemostatic treatments.

**Electronic supplementary material:**

The online version of this article (10.1186/s13063-018-2900-4) contains supplementary material, which is available to authorized users.

## Background

Acute severe haemorrhage is a common complication of injury, childbirth, surgery, gastrointestinal pathologies and other medical conditions. Regardless of the cause, serious bleeding often has similar pathophysiological consequences, such as those mediated by hypovolemic shock. Although efforts to achieve haemostasis depend on the site of bleeding, treatments to support coagulation and maintain vital organ perfusion are not site specific and are often included in generic major haemorrhage protocols [1]. For these reasons, clinical trials assessing the risks and benefits of haemostatic treatments often evaluate the same patient outcomes regardless of the cause or site of bleeding [2–4].

Outcomes in clinical trials should be relevant to patients, amenable to unbiased assessment and have the potential to be influenced by the trial treatment. Because trial results inform the care of different patients, in different places and at different times, we must also consider generalisability when selecting outcomes. We use data from large randomised placebo-controlled trials of tranexamic acid in acute severe bleeding (postpartum, traumatic and gastrointestinal) to assess the extent to which commonly used outcome measures meet these criteria. The CRASH-2 trial is a randomised trial of tranexamic acid in 20,211 trauma patients with, or at risk of, significant bleeding, within 8 h of injury [5]. The WOMAN trial is a randomised trial of tranexamic acid in 20,060 women with postpartum haemorrhage [6]. The HALT-IT trial is a randomised trial of tranexamic acid in 12,000 patients with significant gastrointestinal bleeding [7]. The methods are described in detail elsewhere [5–7]. The HALT-It trial is ongoing, but blinded data on 8699 patients are used in these analyses.

## All-cause or cause-specific mortality?

Because death is important to patients, easy to quantify and may be affected by treatment, it is an important outcome measure in clinical trials in life-threatening bleeding. All-cause mortality is unequivocal and avoids any uncertainties in correctly ascribing the cause of death [8, 9]. Nevertheless, all-cause mortality has important disadvantages as an outcome measure in clinical trials [8, 10, 11].

### Lower power for all-cause mortality

Many deaths following acute severe haemorrhage are due to patient comorbidities or complications rather than the failure to control bleeding. For example, patients with acute upper gastrointestinal bleeding secondary to gastric cancer may survive the acute bleed but die from cancer within the trial follow-up period. Although a haemostatic treatment might affect deaths from bleeding or thrombosis, it would be unrealistic to expect similar, if any, effects on other causes of death. This can lead to low power for all-cause mortality, even in large trials [11].

**Fig. 1:**
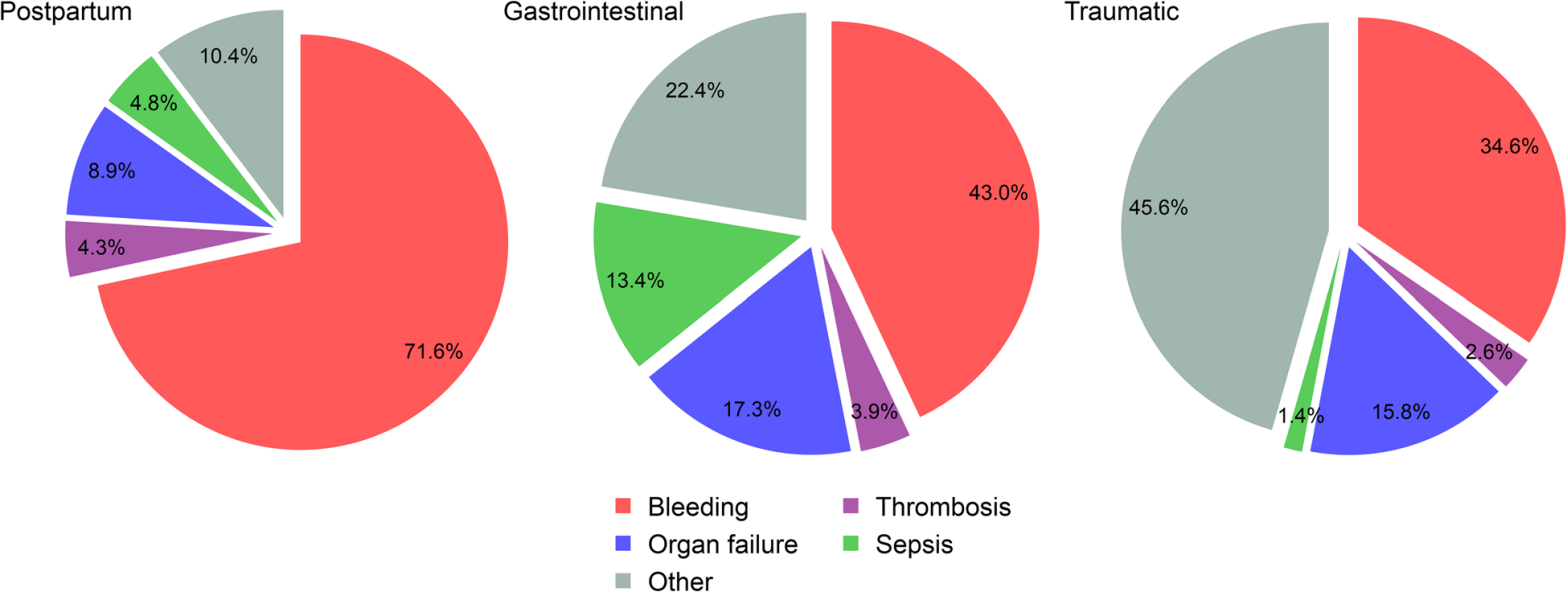
Primary cause of death by site of acute severe haemorrhage. Other causes of death in traumatic haemorrhage include head injury (39.8%). Other causes of death in gastrointestinal haemorrhage include cancer (10.3%) and liver disease (2.3%). Other causes of death in postpartum haemorrhage include eclampsia (2.1%) and pulmonary oedema (1.5%)

**Fig. 2:**
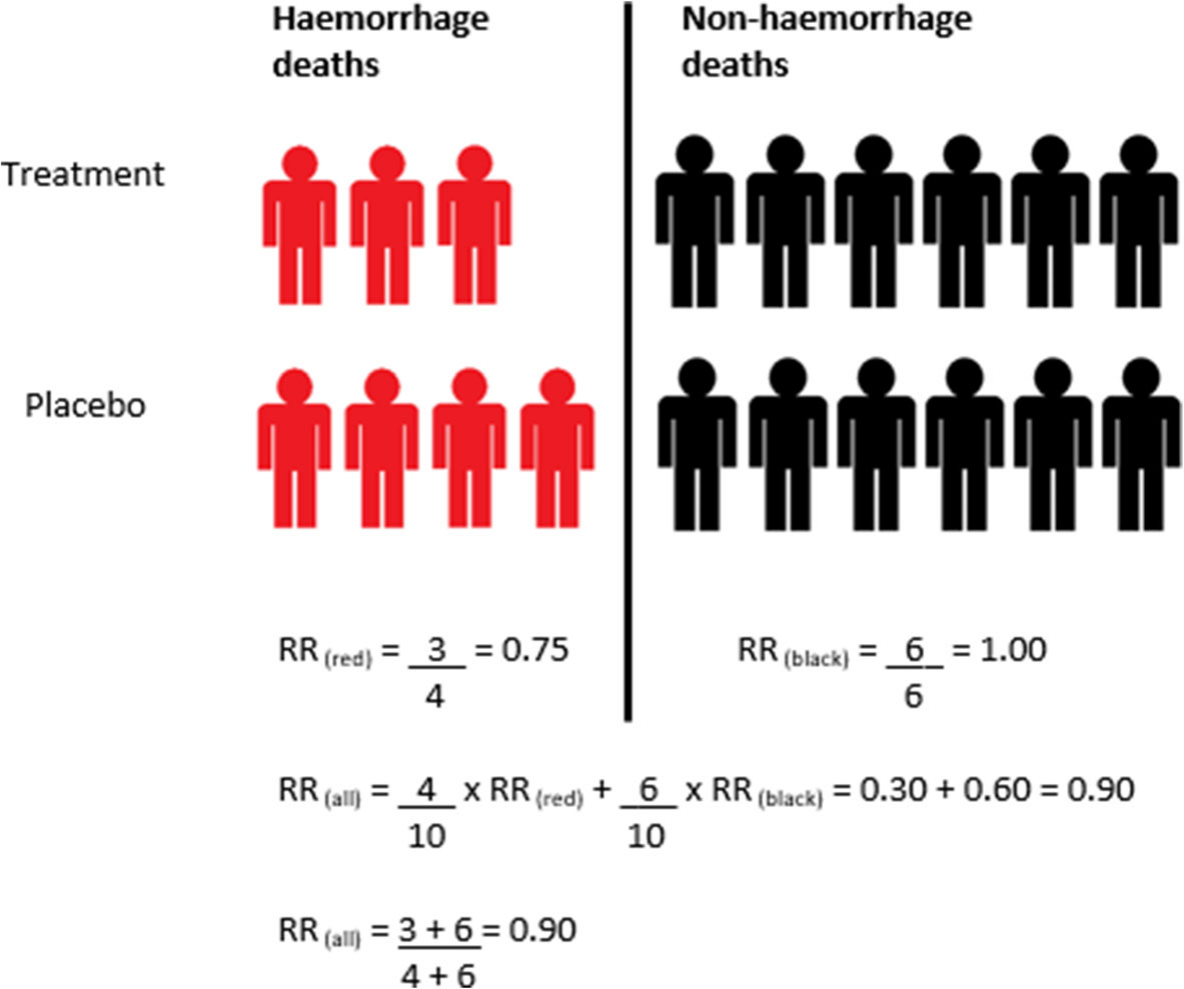
Hypothetical model of the effect of a haemostatic treatment on all-cause and cause-specific mortality. The treatment reduces the risk of death due to bleeding by 25% (relative risk (*RR*) = 0.75) but has no effect on non-bleeding deaths (RR = 1.00). The effect on all-cause mortality (RR = 0.90) is a weighted average of the effect on cause-specific deaths, weighted according to the relative contributions of each cause. Assuming the same number of patients in each trial arm, the RR can also be calculated as the ratio of events in the treatment and placebo groups

Table 1 shows the causes of death in patients with postpartum, traumatic and gastrointestinal haemorrhage. Although bleeding is important in each scenario, the contribution of non-bleeding deaths to all-cause mortality varies between 30 and 65% (see Fig. 1). Since there is usually no reason why a haemostatic intervention would reduce non-bleeding deaths, the effect of the intervention on all-cause mortality will be smaller than the effect on death from bleeding. More precisely, the effect on all-cause mortality will be a weighted average of the effects on specific causes of death, weighted according to their relative contribution to all-cause mortality (see Fig. 2). If non-bleeding deaths are common and are unaffected by the trial treatment, the dilution will be considerable, and a trial would have low power for all-cause mortality, even if there was a significant reduction in bleeding deaths. Sample size depends inversely on the square of the effect size, so a bigger sample is needed to achieve the same power for all-cause mortality as for cause-specific mortality [12]. For example, four times as many patients are needed if only 50% of deaths are due to the cause being affected by the trial medication (i.e. bleeding), and nine times as many are needed if a third of deaths are due to the relevant cause.

**Table 1:** Cause of death and time from randomisation to death in postpartum, gastrointestinal and traumatic haemorrhage

Cause of death	Postpartum haemorrhage		Gastrointestinal haemorrhage		Traumatic haemorrhage^b^	
	*N* = 20,021		*N* = 8,699		*N* = 20,127	
	*n* (%)	Days (hours)	*n* (%)	Days (hours)	*n* (%)	Days (hours)
Bleeding	346 (1.7)	0 (5)	350 (4.0)	1 (28)	1063 (5.3)	0 (10)
Thrombosis^a^	21 (0.1)	0 (11)	32 (0.4)	4 (94)	81 (0.4)	4 (88)
Organ failure	43 (0.2)	2 (47)	141 (1.6)	5 (127)	486 (2.4)	3 (83)
Sepsis	23 (0.1)	5 (118)	109 (1.3)	6 (140)	44 (0.2)	9 (219)
Other	50 (0.2)	1 (13)	182 (2.1)	5 (114)	1402 (7.0)	1 (35)
All-cause	483 (2.4)	0 (7)	814 (9.4)	3 (66)	3076 (15.3)	1 (22)

Time to death is the median time from randomisation to death in days and hours

^a^Includes stroke, myocardial infarction and pulmonary embolism

^b^Time to death estimated using date and time of randomisation and date of death

### Important safety signals may be obscured in all-cause mortality

**Fig. 3:**
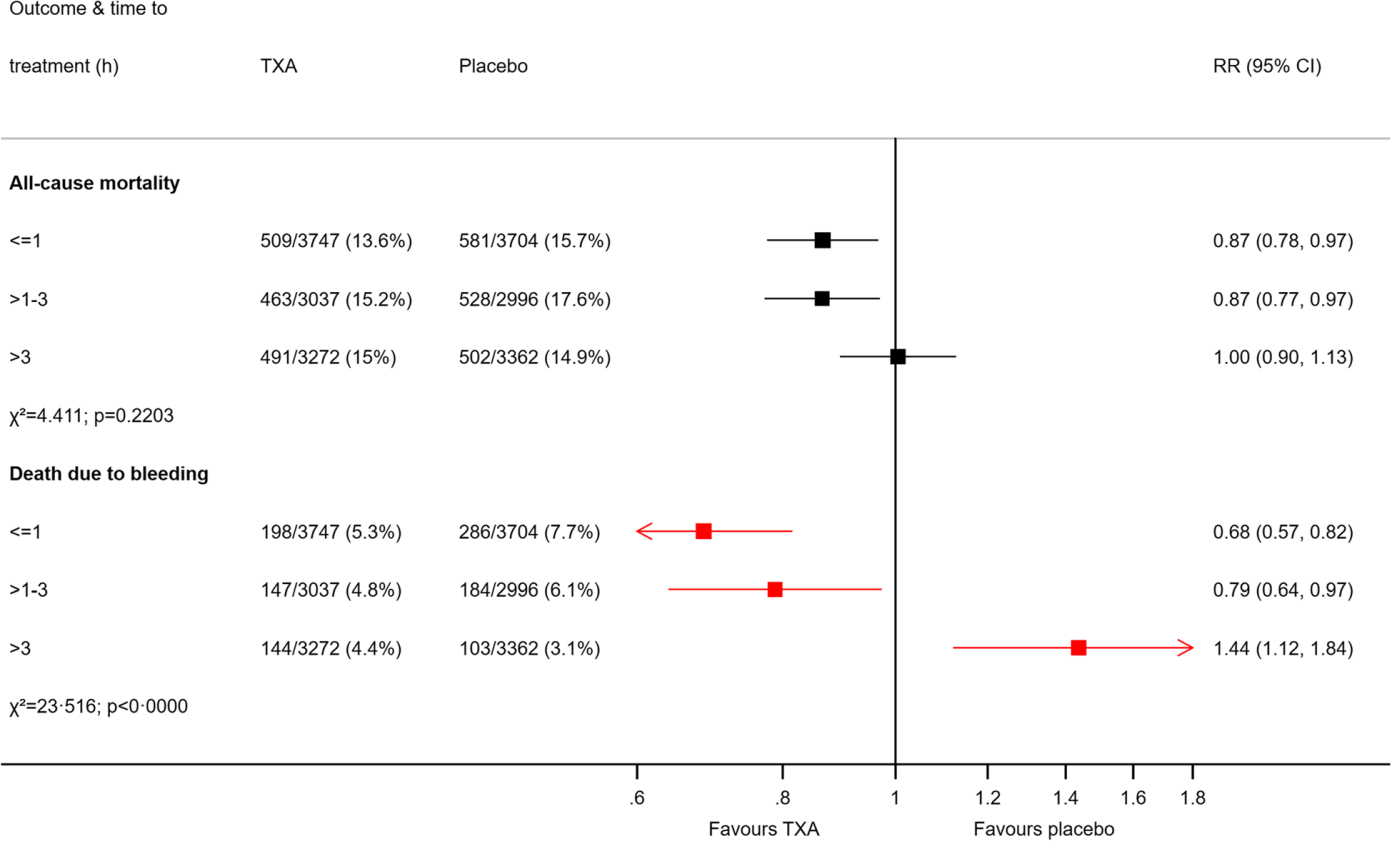
Effect of tranexamic acid on all-cause mortality and death due to bleeding in traumatic haemorrhage by time to treatment

Due to the dilution from deaths unaffected or reduced by the trial treatment, all-cause mortality can also obscure important harmful effects, which are typically rarer and also need to be considered on a cause-specific basis [10, 11]. For example, there is strong evidence that the effect of tranexamic acid on bleeding deaths varies by time to treatment, with a 10% decrease in survival benefit for every 15-min delay [13]. Treatment given more than 3 h from bleeding onset is ineffective and possibly harmful [14]. However, this strong time-to-treatment interaction is obscured in analyses of all-cause mortality (see Fig. 3). For the same reason, we must assess separately any potential adverse effects of haemostatic treatments (e.g. increased risk of thrombotic deaths). These are often missed in all-cause endpoints due to the effect being swamped and obscured by other causes of death. Risk-benefit decisions in individuals also require separate assessment of benefits and harms because the baseline risks vary between patients. A haemostatic drug might reduce all-cause mortality in a young patient at low baseline risk of thrombosis but not in an older patient with cardiovascular comorbidity.

### Generalisability

Because the relative contribution of different causes of death varies within and between patient populations, all-cause mortality is not generalisable. For example, in the CRASH-2 trial, bleeding accounted for 60% of deaths in patients with penetrating trauma compared to 25% of deaths in patients with blunt trauma. There was a substantial reduction in death due to bleeding with tranexamic acid, with no heterogeneity by type of injury, but no reduction in non-bleeding deaths (see Table 2). Consequently, although the effect of tranexamic acid on death due to bleeding is essentially the same in blunt and penetrating injury, it will have a larger effect on all-cause mortality in populations where penetrating trauma is common.

**Table 2:** Effect of early tranexamic acid on all-cause, cause-specific and time-specific mortality in postpartum and traumatic haemorrhage

Cause/time of death	Postpartum haemorrhage		Traumatic haemorrhage^b^		All	
	(*N* = 14,923)		(*N* = 13,484)		(*N* = 28,407)	
	RR (95% CI)	*p* value	RR (95% CI)	*p* value	RR (95% CI)	*p* value
All-cause	0.83 (0.66–1.04)	0.099	0.87 (0.80–0.94)	< 0.001	0.86 (0.80–0.93)	< 0.001
Cause-specific						
Haemorrhage	0.69 (0.53–0.90)	0.007	0.72 (0.63–0.83)	< 0.001	0.72 (0.63–0.81)	< 0.001
Thrombosis^a^	1.15 (0.39–3.42)	0.803	0.56 (0.31–0.99)	0.043	0.65 (0.39–1.07)	0.090
Other	1.35 (0.84–2.15)	0.213	0.99 (0.89–1.10)	0.867	1.01 (0.90–1.12)	0.908
Time-specific (hours from randomisation)						
< 48	0.74 (0.58–0.95)	0.015	0.83 (0.75–0.91)	< 0.001	0.81 (0.74–0.89)	< 0.001
≥ 48	1.81 (0.92–3.55)	0.080	0.94 (0.81–1.10)	0.457	0.98 (0.85–1.15)	0.844

Includes patients treated within 3 h of delivery/injury only

^a^Includes stroke, myocardial infarction and pulmonary embolism

^b^Time of death estimated using time and date of randomisation and date of death

*CI* confidence interval, *RR* relative risk

### Misclassification of cause of death

The main concern with cause-specific mortality is that cause of death is determined subjectively and can be misclassified [15, 16]. For blinded trials, any misclassification would be unrelated to treatment allocation and so will not introduce bias. Although misclassification of cause of death might dilute the effect of the treatment on cause-specific mortality, the power of a trial to detect a slightly diluted measure of the relevant (generalisable) outcome should be higher than that for all-cause mortality. When cause of death is ascertained by methods with very low sensitivity and specificity, the power of a trial to detect a treatment effect on cause-specific and all-cause mortality may be similar [17]. This also occurs when most of the deaths are due to the cause under study. Although independent, blinded event adjudication by an endpoint review committee is thought to provide an unbiased and systematic method for evaluating causes of death in clinical trials, there is little empirical evidence that this has any substantial effect on trial accuracy [18–20].

## Time-specific mortality

Misclassification of cause of death is a particular concern in un-blinded trials, where knowledge of group allocation might influence decisions about cause of death and introduce bias. Because different causes of death occur at different time intervals from bleeding onset, time-specific mortality can help maintain objectivity whilst avoiding the drawbacks of all-cause mortality. Table 1 shows the time interval between hospital admission and death by cause of death and site of haemorrhage. Most bleeding deaths occur within 48 h of admission, followed by deaths from vascular occlusion and multi-organ failure, with sepsis deaths about 1 week later. Table 2 shows the effect of tranexamic acid on death due to bleeding and death within 48 h of admission in traumatic and postpartum haemorrhage. The results are similar, suggesting deaths within 48 h of admission might be used as a proxy for bleeding deaths in non-blinded trials. Although some misclassification is inevitable, especially if there are many early non-bleeding deaths, misclassification rates should not differ by allocated group.

In some scenarios, re-bleeding is common and can cause death. More than half of patients with liver disease who survive an episode of variceal bleeding will re-bleed within a year, and one fifth of these patients will die [21]. Re-bleeding also occurs after spontaneous intracranial haemorrhage. A patient enrolled in a trial of a haemostatic agent may survive the initial bleed but die from re-bleeding during the follow-up. Depending on the duration of the trial treatment and the half-life of the drug, it may be unrealistic to expect a treatment given for the initial bleed to influence re-bleeding deaths many days or weeks later, and the inclusion of these re-bleeding deaths could dilute the effect. In this situation, cause-specific mortality within a specified interval of the index bleed may be more appropriate.

Time-specific death can also be a useful endpoint when cause-specific mortality cannot be assessed. For example, in patients with spontaneous and traumatic intracranial bleeding it is difficult to determine the pathophysiological process (e.g. haemorrhage, oedema, infarction) leading to death [22–26]. However, because most intracranial bleeding occurs within hours of symptom onset with significant haematoma expansion being rare after 24 h, early deaths are more likely to be affected by a haemostatic agent than late deaths [24, 25]. The TICH-2 trial of tranexamic acid in spontaneous intracranial bleeding found a significant reduction in deaths within 7 days with less haematoma expansion, but no reduction in death at 90 days [27]. Whilst this cannot be taken as evidence of efficacy, it suggests the need for larger adequately powered trials.

## Surgical intervention and blood transfusion as outcome measures

Surgical interventions to control bleeding and receipt of blood transfusion are common outcome measures in trials of haemostatic treatments. At first sight, they appear to satisfy our three criteria. Patients would prefer not to undergo surgery or receive allogenic blood; these outcomes are well documented; and both could be reduced by an effective haemostatic treatment. However, whilst surgery and transfusion may be suitable in bleeding prevention trials, they are less appropriate in treatment trials, where urgent treatment is critical and trial recruitment can often take second place. The activation of major haemorrhage protocols and decisions regarding established interventions are likely to happen before or around the same time as the administration of a trial treatment. Indeed, trials in elective surgery show that tranexamic acid reduces blood transfusion by about one third, whereas there was no effect on transfusion in trials of tranexamic acid for the treatment of postpartum or traumatic haemorrhage [5, 6, 28].

Death or hysterectomy was the primary outcome in the WOMAN trial of tranexamic acid treatment for postpartum haemorrhage. However, during the trial the investigators noticed that the decision to conduct an emergency peripartum hysterectomy was often made at the time of randomisation. For example, in response to life-threatening bleeding, a clinician might elect to do a hysterectomy and then enrol the woman into the trial. Although tranexamic acid might prevent death in these women, it could not prevent hysterectomy. In response, investigators increased the sample size from 15,000 to 20,000 patients to provide enough power to detect a reduction in bleeding deaths. On the other hand, there was a substantial reduction in re-operation to control bleeding with tranexamic acid. Unlike hysterectomy, the decision to re-operate is made after randomisation and so could be influenced by tranexamic acid.

Similarly, the receipt of blood transfusion after randomisation is mostly determined by blood lost prior to randomisation (see Additional file 1: Tables S1 and S2). Major haemorrhage protocols triggered by estimated blood loss or blood pressure on admission (i.e. before randomisation) largely dictate the amount of blood transfused through generic blood protocols, which specify the number and ratio of blood components transfused. Although administered post-randomisation, transfusions given in response to presenting clinical signs and symptoms caused by blood lost before randomisation cannot be affected by the trial treatment, and this will dilute the treatment effect. For example, if we assume 80% of post-randomisation transfusions are given for blood lost before randomisation (relative risk (RR) = 1.00) and 20% are given for blood lost afterwards (RR = 0.70), the overall effect on transfusion, the weighted average of the two, will be severely diluted (RR = 0.94). It is also important to bear in mind that in some countries receipt of transfusion does not reflect blood loss due to blood shortages. Finally, if the trial treatment improves survival, there will be a greater opportunity to receive a transfusion in the treatment arm. For these reasons, we should not expect a substantial reduction in the need for transfusion in trials of treatments for acute severe haemorrhage.

## Conclusions

When a patient has acute severe bleeding, time is of the essence, and urgent care inevitably takes priority over the administration of a trial treatment. As such, blood transfusion and surgery are often planned before or at the time of randomisation, and so cannot be prevented by the trial treatment. Indeed, the only patient outcome that can be clearly established as following the administration of the trial treatment is death. However, because many deaths in patients with acute severe bleeding are from comorbidities that may be unaffected by the trial treatment, even large trials will have low power to detect changes in all-cause mortality. Both benefit and harm can be obscured in all-cause mortality, and because the relative contributions of different causes of death vary within and between patient populations, all-cause mortality is not generalisable. Cause-specific mortality, such as death due to bleeding or thrombosis, avoids the drawbacks of all-cause mortality. Although assigning cause involves judgement, this will not cause bias in blind placebo-controlled trials. Time-specific mortality can be a proxy for cause in un-blinded trials or when cause of death cannot be assessed. Core outcome sets for trials evaluating treatments for life-threatening bleeding [29, 30] should consider the results of these analyses.

## Additional file

Additional file 1: Supplementary data analyses. This file provides two tables showing the relationship between baseline characteristics and blood transfusion in postpartum and traumatic haemorrhage. (DOCX 27 kb)

## Abbreviations

CRASH-2: Clinical Randomisation of an Antifibrinolytic in Significant Haemorrhage
HALT-IT: Haemorrhage Alleviation with Tranexamic Acid - Intestinal System
WOMAN: World Maternal Antifibrinolytic
