# Supplementary material for: Outcome measures in clinical trials of treatments for acute severe haemorrhage
Source: Trials. 2018 Oct 1;19:533. doi: 10.1186/s13063-018-2900-4 (PMC6167881; doi:10.1186/s13063-018-2900-4)
Supplement: Supplementary file 1 — Supplementary data analyses. This file provides two tables showing the relationship between baseline characteristics and blood transfusion in postpartum and traumatic haemorrhage. (DOCX 27 kb) [file 13063_2018_2900_MOESM1_ESM.docx]

**Additional file 1 – Supplementary data analyses**

Table S1: Relationship between blood transfusion and baseline characteristics in postpartum haemorrhage

Table S2: Relationship between blood transfusion and baseline characteristics in traumatic haemorrhage

**Table S1. Relationship between blood transfusion and baseline characteristics in postpartum haemorrhage**

| **Baseline characteristic** | **Blood transfusion** | | | |  |
| --- | --- | --- | --- | --- | --- |
|  | Crude odds ratio (95% CI) | P-value* | Adjusted odds ratio (95% CI) | P-value** | |
| **Age** |  |  |  |  | |
| 14-19 | 1.00 | <0.0001 | 1.00 | 0.5373 | |
| 20-24 | 1.15 (1.00-1.32) |  | 0.96 (0.82-1.13) |  | |
| 25-29 | 1.39 (1.22-1.59) |  | 0.96 (0.82-1.12) |  | |
| 30-34 | 1.65 (1.45-1.89) |  | 0.97 (0.83-1.13) |  | |
| 35+ | 2.09 (1.81-2.40) |  | 1.04 (0.88-1.23) |  | |
| **Estimated blood loss (ml)** |  |  |  |  | |
| <1000 | 1.00 | <0.0001 | 1.00 | <0.0001 | |
| 1000-1499 | 2.85 (2.65-3.06) |  | 2.65 (2.45-2.86) |  | |
| 1500-1999 | 6.50 (5.95-7.11) |  | 5.99 (5.42-6.63) |  | |
| 2000+ | 25.49 (22.10-29.40) |  | 22.07 (18.95-25.71) |  | |
| **Systolic blood pressure** |  |  |  |  | |
| >=90 | 1.00 | <0.0001 | - | - | |
| 76-89 | 2.09 (1.90-2.30) |  |  |  | |
| <=75 | 3.35 (2.98-3.77) |  |  |  | |
| **Cause of haemorrhage** |  |  |  |  | |
| Uterine atony | 1.00 | <0.0001 | 1.00 | <0.0001 | |
| Placenta praevia/accreta | 3.56 (3.18-3.99) |  | 2.03 (1.78-2.32) |  | |
| Surgical tears/trauma | 1.39 (1.29-1.49) |  | 1.20 (1.10-1.31) |  | |
| Other | 2.15 (1.92-2.42) |  | 1.61 (1.41-1.84) |  | |
| Unknown | 1.01 (0.79-1.30) |  | 0.95 (0.70-1.29) |  | |
| **Type of delivery** |  |  |  |  | |
| Vaginal | 1.00 | <0.0001 | 1.00 | 0.0034 | |
| C-section | 2.18 (2.04-2.32) |  | 1.13 (1.04-1.23) |  | |
| **Place of delivery** |  |  |  |  | |
| In hospital | 1.00 | <0.0001 | 1.00 | <0.0001 | |
| Out of hospital | 3.55 (3.20-3.93) |  | 3.20 (2.85-3.58) |  | |
| **Uterotonics** |  |  |  |  | |
| Yes | 1.00 | <0.0001 | - | - | |
| No | 3.55 (2.62-4.79) |  |  |  | |
| Unknown | 2.87 (2.32-3.56) |  |  |  | |
| **Treatment group** |  |  |  |  | |
| Placebo | 1.00 | 0.9067 | - | - | |
| TXA | 1.00 (0.95-1.06) |  |  |  | |
| **Country income level** |  |  |  |  | |
| High | 1.00 | <0.0001 | 1.00 | <0.0001 | |
| High-middle | 0.68 (0.53-0.88) |  | 1.60 (1.20-2.14) |  | |
| Low-middle | 2.79 (2.34-3.32) |  | 4.22 (3.44-5.16) |  | |
| Low | 1.17 (0.98-1.41) |  | 1.87 (1.51-2.31) |  | |
| *chi-squared test; **likelihood ratio test | | |  |  | |

**Table S2. Relationship between blood transfusion and baseline characteristics in traumatic haemorrhage**

| **Baseline characteristic** | **Blood transfusion** | | | |  |
| --- | --- | --- | --- | --- | --- |
|  | Crude odds ratio (95% CI) | P-value* | Adjusted odds ratio (95% CI) | P-value** |  |
| **Sex** |  |  |  |  |  |
| Male | 1.00 | 0.0021 | 1.00 | 0.0653 |  |
| Female | 1.12 (1.04-1.21) |  | 1.08 (1.00-1.17) |  |  |
| **Age** |  |  |  |  |  |
| <25 | 1.00 | 0.4935 | - | - |  |
| 25-34 | 1.05 (0.97-1.13) |  |  |  |  |
| 35-44 | 1.06 (0.98-1.15) |  |  |  |  |
| >44 | 1.04 (0.96-1.12) |  |  |  |  |
| **Systolic blood pressure** |  |  |  |  |  |
| >=90 | 1.00 | <0.0001 | 1.00 | <0.0001 |  |
| 76-89 | 2.62 (2.42-2.84) |  | 2.60 (2.40-2.82) |  |  |
| <=75 | 3.83 (3.52-4.18) |  | 3.60 (3.30-3.93) |  |  |
| **Heart rate** |  |  |  |  |  |
| 77-91 | 1.00 | <0.0001 | - | - |  |
| <77 | 1.25 (1.11-1.40) |  |  |  |  |
| 92-107 | 1.37 (1.26-1.50) |  |  |  |  |
| >107 | 2.54 (2.34-2.75) |  |  |  |  |
| **Respiratory rate** |  |  |  |  |  |
| 10-29 | 1.00 | <0.0001 | - | - |  |
| >29 | 1.72 (1.58-1.86) |  |  |  |  |
| <10 | 2.31 (1.82-2.95) |  |  |  |  |
| **Capillary refill time (seconds)** |  |  |  |  |  |
| <=2 | 1.00 | <0.0001 | - | - |  |
| 3-4 | 1.29 (1.21-1.37) |  |  |  |  |
| >4 | 2.18 (2.01-2.38) |  |  |  |  |
| **Glasgow Coma Scale** |  |  |  |  |  |
| Mild | 1.00 | <0.0001 | 1.00 | <0.0001 |  |
| Moderate | 1.49 (1.37-1.62) |  | 1.23 (1.13-1.34) |  |  |
| Severe | 1.70 (1.58-1.83) |  | 1.43 (1.32-1.55) |  |  |
| **Injury type** |  |  |  |  |  |
| Blunt | 1.00 | 0.0139 | 1.00 | 0.0003 |  |
| Penetrating | 0.93 (0.88-0.99) |  | 0.88 (0.82-0.94) |  |  |
| **Treatment group** |  |  |  |  |  |
| Placebo | 1.00 | 0.2022 | - | - |  |
| TXA | 0.96 (0.91-1.02) |  |  |  |  |
| **Country income level** |  |  |  |  |  |
| High | 1.00 | <0.0001 | 1.00 | <0.0001 |  |
| High-middle | 0.24 (0.18-0.31) |  | 0.26 (0.20-0.33) |  |  |
| Low/low-middle | 0.19 (0.18-0.31) |  | 0.21 (0.16-0.27) |  |  |
| *chi-squared test; **likelihood ratio test | | | | | |
